# Resignation of Prof. John C. Warren

**Published:** 1847-04

**Authors:** 


*Resignation of Prof. John C. Warren.*—We perceive by the Boston Medical and Surgical Journal that Dr. John C. Warren, who has for many years occupied, with distinguished honor, the chair of Anatomy and Operative Surgery in the Massachusetts Medical College, has recently tendered his resignation of office.—

Who is to be his successor is not yet announced. It is stated that Dr. W. will continue his connection with the Massachusetts General Hospital. Dr. W. will carry with him in his retirement from the duties of a medical teacher, sentiments of grateful esteem from all of the many who have listened to his valuable instructions.

